# Communicating with young children who have a parent dying of a life-limiting illness: a qualitative systematic review of the experiences and impact on healthcare, social and spiritual care professionals

**DOI:** 10.1186/s12904-022-01007-1

**Published:** 2022-07-12

**Authors:** Lasitha M. Wickramasinghe, Zhi Zheng Yeo, Poh Heng Chong, Bridget Johnston

**Affiliations:** 1 HCA Hospice, 705 Serangoon Road, #03-01 Block A @ Kwong Wai Shiu Hospital, Singapore, 328127 Singapore; 2 School of Medicine, Dentistry and Nursing, University of Glasgow, Glasgow, G12 8QQ UK

**Keywords:** Palliative care, Healthcare professional, Healthcare worker, Terminally ill, Dying, End of life, Young children, Dependent children

## Abstract

**Background:**

Healthcare professionals play a key role in interacting with children who have a parent with a life-limiting illness. While playing such a role can be challenging, not much is known about how such interactions impact these professionals and affect their ability to render support.

**Methods:**

Four databases were searched with the intention to conduct a qualitative systematic review. Articles were selected based on pre-determined inclusion and exclusion criteria. Their quality was assessed using the tool "Standard Quality Assessment Criteria for Evaluating Primary Research Papers from a Variety of Fields”. Findings were analysed using thematic analysis techniques outlined by Thomas and Harden as well as Sandelowski and Barroso. Review was registered with the Review Registry database.

**Results:**

Three themes emerged – healthcare professionals’ discomfort; their assumptions and actions; and potentiating workplace factors. The discomfort had several dimensions: fear of making a situation worse, concern of not being able to cope with emotionally charged situations, and internal conflict that arose when their values clashed with family dynamics.

**Conclusion:**

Healthcare professionals’ sense of discomfort was very pronounced. This discomfort, together with their assumptions, could impact their ability to support children. The organisation played an important role, which was reflected in the work culture, workflow and ability to collaborate with other agencies involved in supporting children. The discomfort was mitigated by having more professional experience, workplace support systems and training on communicating with children. It was apparent that the individual professional did not work alone when supporting children but alongside others within an organisation. As such, issues raised in this review will benefit from multi-faceted solutions.

**Supplementary Information:**

The online version contains supplementary material available at 10.1186/s12904-022-01007-1.

## Background

Supporting young children when their parent has a life-limiting illness can be challenging. Healthcare providers play a key role in interacting with children in this situation, but we do not know how such interactions impact these professionals and individual perceptions on their ability to render support. Only by understanding the healthcare providers’ concerns can we address them, thus enabling them to provide more effective support.

Palliative care for seriously ill parents with dependent children is increasing, as people live longer [1] and have children later [2], coupled with increasing incidence and morbidity of chronic diseases [3]. Worldwide, over 56.8 million people are estimated to require palliative care every year, out of which 27% are aged 50–69 and around 26% aged 20–49 [4]. While specific data on number of children who had lost a parent to life-limiting illness is not available, extrapolation from mortality and census data showed that in 2015, 23,600 parents died in the UK, leaving behind around 41,000 dependent children aged 0–17 [5]. Young children do understand dying and perceive the implications of their parent’s terminal illness [6, 7]; those left in the dark may experience adverse psychological and emotional development [6]. Despite this, parents continue to shield young children from reality out of a desire to protect them [8]. Terminal illness, rather like an unwanted guest, causes a great deal of upheaval to the young family. At a tumultuous time, parents often struggle to support their children emotionally, and so healthcare, social and spiritual care professionals (from here on referred to as “professionals”) may be expected to fill this gap [8].

Evidence is lacking about the impact on professionals when providing emotional support to children, which is surprising, given the important role they play here. Existing research are heterogeneous; designs primarily qualitative, use different methodologies and cross diverse disciplines. We performed a systematic review to appreciate current understanding and to identify knowledge gaps. Our review question was “What are the experiences and impact on healthcare, social and spiritual care professionals when communicating with young children who have a parent dying of a life-limiting illness?”.

## Methods

A qualitative systematic review was conducted following the approaches of Thomas and Harden as well as Sandelowski and Barroso, as an early scoping review revealed mostly qualitative studies [9, 10].

### Search process

**Fig. 1:**
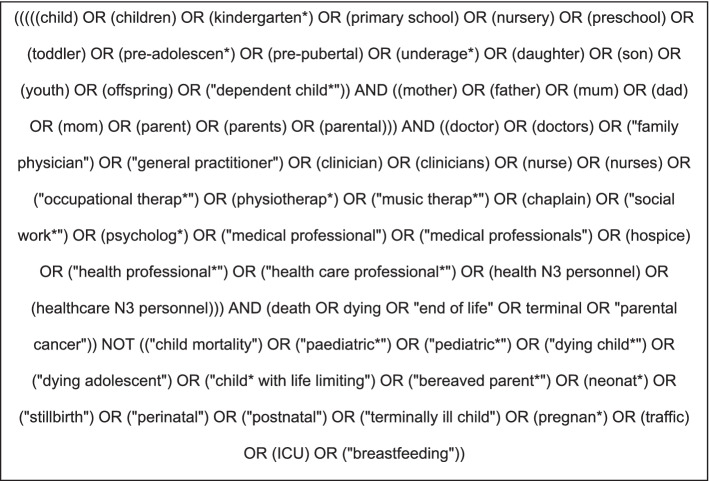
Search strategy used in PubMed

Four databases (PubMed, CINAHL, Embase and PsycINFO) were searched for peer reviewed literature published 2000–2020. Keywords, MeSH terms (PubMed) and equivalent (for the other databases) were utilised. A sample of our search strategy is illustrated in Fig. 1.

More articles were identified by backward and forward chaining, supplemented by hand searching where applicable. This review was registered with the Review Registry on April 27^th^, 2021 [11].

### Study selection

Results from searches were combined using EndNote, and duplicates removed. The first author (LMW) initially screened titles and abstracts with reference to review question. Two authors (LMW and ZZY) identified articles to read in full using the inclusion and exclusion criteria (Table 1).

**Table 1:** Inclusion and Exclusion Criteria

Inclusion Criteria	Exclusion Criteria
Children 12 years and below with a parent dying of a life-limiting illness (including but not limited to cancers, end organ disease/failure and neurological conditions)	Children aged 13 years and above or already bereaved. Dying patient is a sibling/child, or an adult on whom the child is not dependent. Potential or final cause of death not due to life-limiting illness (e.g., suicide, drowning, road traffic accident)
Empirical studies including perspectives from health, social and spiritual care professionals working in a clinical capacity where they support terminally ill patients (including but not limited to Palliative Medicine, Oncology and Geriatrics). Such professionals include doctors, nurses, social workers, allied health, counsellors and chaplains	Studies that exclusively include stakeholders apart from health professionals, e.g., child, parent. Studies where patients received terminal or end-of-life care in Intensive Treatment Unit/ Accident and Emergency Department
Articles published in peer-reviewed journals from year 2000 onwards	Articles published before year 2000 in publications that are not peer-reviewed
Articles written and published in English, including English translations of articles written in other languages	Articles with no official English translation
Includes qualitative and quantitative studies of any design	Excludes opinion pieces, editorials, reviews and other grey literature

Given that children across age ranges have different levels of understanding of death and dying, we chose to focus on interactions with children aged 12 years and below. In articles where professionals had interacted with minors of differing ages, only data referring to children fitting the inclusion criteria were analysed.

### Quality assessment

Articles shortlisted were assessed using "Standard Quality Assessment Criteria for Evaluating Primary Research Papers from a Variety of Fields” [12] that accommodated inherent study heterogeneity. The tool was comprehensive; ten criteria appraised a study’s research question, study design and context, sampling strategy, data collection and analysis, conclusions and researcher reflexivity. No cut-off score had been specified by the original authors. We only included articles that scored above 12 out of 20 (above 60%) to optimise robustness of data synthesised. To minimise bias, articles were independently assessed by two authors (LMW and ZZY), with a third author (PHC) arbitrating if needed.

### Data extraction and synthesis

First and second order findings under results and discussion sections respectively extracted from each article were analysed thematically as outlined by Thomas and Harden [10]. The iterative process consisted of three steps:

1. Creating free codes: Findings from primary studies were coded line-by-line using NVivo version 12. The free codes facilitated later translation of concepts between studies.
2. Grouping codes into descriptive themes: Codes were analysed further for meaning, and then reorganised into related thematic categories.
3. Creating analytical themes: Each category created was examined and compared to others. Similar categories were analysed, re-interpreted and grouped to create higher-level constructs (analytical themes). These themes eventually generated conclusions that consolidated findings from individual studies.

Analytical approaches outlined by Sandelowski and Barroso [9] complemented steps above at later stages of data analysis; a table listing all selected studies and pertinent findings across key elements facilitated cross-study comparisons, enabling the uncovering of trends or patterns. Data analysis deepened through multiple discussions between this review’s authors, reflecting on anecdotal experiences working with such children. Final conclusions were depicted in a conceptual model that aimed to depict relevant experiences of professionals and the impact on them gleaned from contemporary literature.

## Results

### Review process

**Fig. 2:**
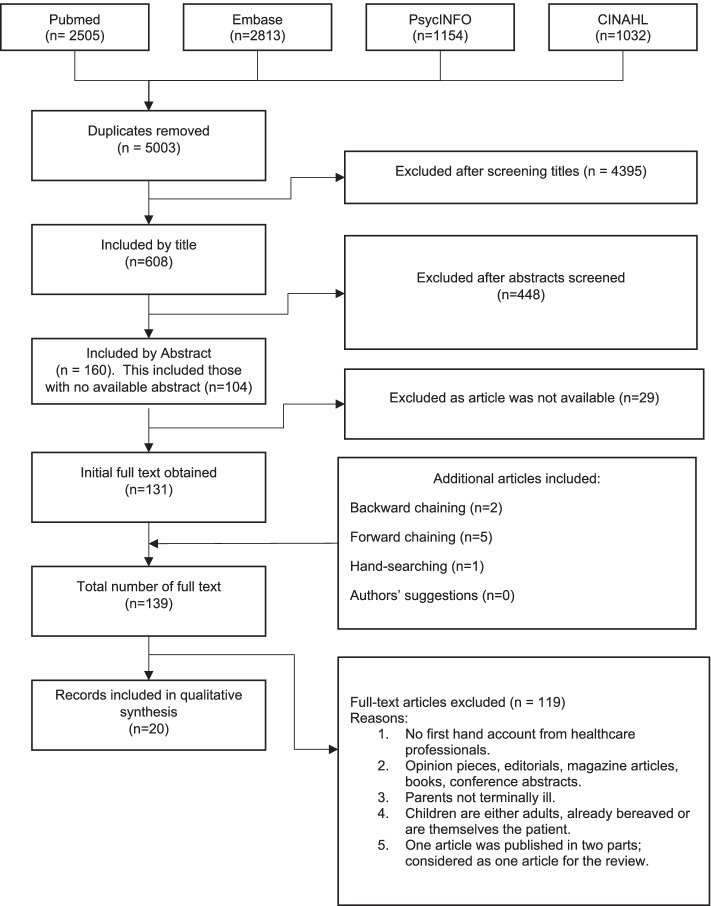
PRISMA Diagram

Figure 2 shows the PRISMA diagram. 7504 articles were first identified. 20 articles were finally selected for synthesis (Additional file 1). Macpherson et al. published an article in two parts and was counted as one article [13, 14]. All included articles met quality assessment criteria set a priori [12] and were deemed relevant to our review as determined by Sandelowski and Barroso [9].

Half the 20 articles reviewed were from UK, and the remainder mostly from Scandinavian countries. There was one article from Japan, and two from Australia. Participants were majority from tertiary hospital settings. A few studies focused on professionals working in hospices or the community. Findings from all selected studies were qualitative in nature, primarily obtained via focus groups or individual interviews. Nurses made up most of the participants. Sample sizes varied from as low as 3 to 32. A study that surveyed staff in adult hospices received 130 replies.

### Themes

**Fig. 3:**
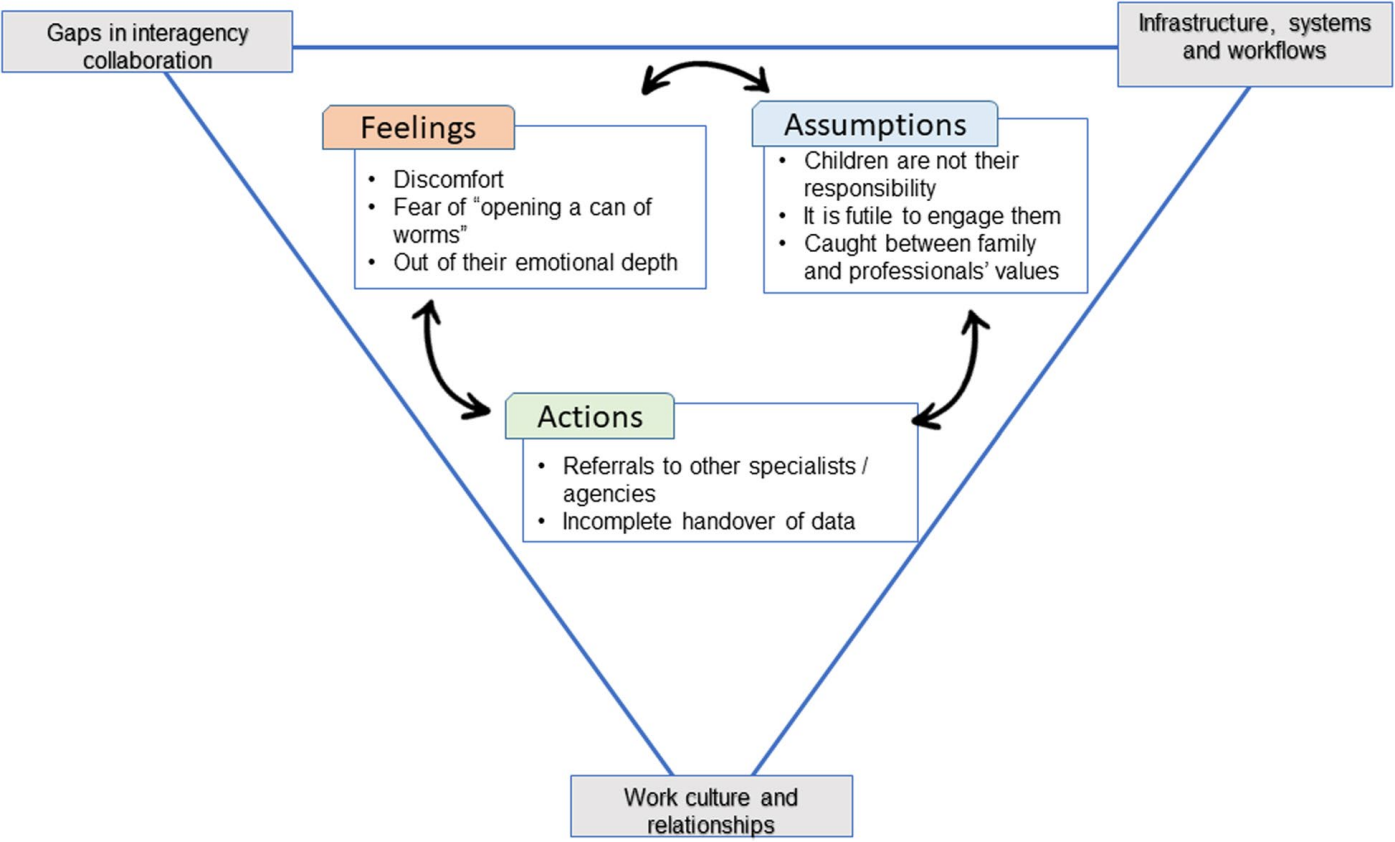
Conceptual Model Showing the Interplay between the Themes

Three overarching analytical themes were developed. They revolved around discomfort on the part of the professional, associated thoughts and assumptions, and concomitant actions taken within individual contexts. A conceptual model was created illustrating the interplay between these themes (Fig. 3) with larger systemic factors as backdrop. These themes are explicated next, using relevant quotations from original empirical studies to substantiate conclusions drawn.

### Discomfort

Professionals experienced a pervasive sense of discomfort, which had several dimensions—fear of opening a can of worms; being caught in between family dynamics and their own values; and feeling out of their emotional depth.

#### Fear of opening a can of worms

Professionals feared any intervention on their part might worsen the situation, rather than improve it. The phrase ‘opening a can of worms’ was cited in several articles [15–17]; it was alluded to by professionals and researchers alike over concerns of potentially complicating a situation [18, 19], and not being able to cope emotionally in the aftermath [16]. They had *“concerns about saying the wrong thing, being perceived as critical of parenting, or the potential to cause distress to children and their families when exploring children’s support needs” *[20]. Words such as death, dying and dead were found to be hard to say, resulting in the use of euphemisms that might have led to further confusion. Those words were felt to sound harsh and hence professionals were not comfortable using them [16]. Some professionals who had no specific training relied on their own experiences or intuition [21]. This was even more challenging for those working with ethnically diverse populations [20]. Others lacked confidence in navigating these conversations [17, 20–23] from perceived lack of experience and knowledge [14], which made them fearful of letting the child down [20].

*“The nurses do not know what is permitted and what the limits are in the encounter with the child. They require concrete advice from, e.g., the counsellor, as well as clinical guidelines to help them to understand how best to encounter the child…. The barrier most frequently cited by nurses is that many of them lack the skills, qualifications and experience to support the child, which leads to reluctance to communicate and interact with her/him.” *[24]

The uncertainty brought on by these concerns led to a desire to avoid such situations [24]. However, professionals often failed to realise that *“this ‘can’, rather than containing worms, often held stories of individual courage and resilience which potentially could provide comfort and support to the family as a whole.” *[16]. This fear that stemmed from perceived inexperience or lack of training was only one dimension of the discomfort faced by the professional.

#### Caught in between family dynamics and their own values

Professionals expressed a sense of internal conflict when encountering young children of their patients. This conflict centred on their desire to respect the parents’ wishes on limited involvement of the child while perceiving they had a duty to prepare the child for their parent’s demise.

On one hand, the professionals empathised and had to respect the patient’s wishes. Being the gatekeeper to the child, professionals were obligated to respect their adult patients’ wishes over how much their child was told about their conditions [24]. However, the professional’s own experience informed them that children cope better when aware of what is going on [21, 25, 26]:

*“From the nurses' perspective, the situations where children were excluded from matters related to a parent's illness and forthcoming death seemed more stressful for the remaining parent, the children and other significant people after the death. The situation became more disorderly and chaotic, and the nurses found it more difficult not being able to control and/ or restore the order in the family. “*[26]

Thus, while professionals empathised with the parents, recognising that they might be having difficulties accepting their prognosis and have the desire to protect their own children from distress, their own perceived duty to the child led to a dilemma that left them feeling powerless [18, 19, 21, 22, 25–27]:

*“Some nurses found it difficult when parents were persistent in their determination to protect their children from matters related to their illness and forthcoming death, and in these cases relied on the parents to take responsibility for their children…. Nurses indicated that they knew that by law, children have the right to professional advice, support and information, but at the same time the nurses knew they could not defy the wishes of the parents.”* [26]

On the other hand, the professionals also saw a patient’s children as autonomous actors outside of the routine care sphere. This then resulted in professionals often having the dilemma of not knowing if they should be promoting more open discourse with children or adopt a non-interfering stance [21–24]. Thus, professionals felt less conflicted when parents permitted them to interact with their children [26, 28]. Where prevented, they overcame this by being present for the parent, thereby indirectly supporting the child [22, 23], as well as identifying members of the wider family network who might be able to support the child [14, 24, 29]. Some chose not to promise hiding information actively from the child when asked to do so by parents [26]:

*“Sometimes nurses were in a dilemma when parents asked them not to tell the truth to the children. Some nurses then chose to respond to the parents that they would not lie to the children if they asked direct questions, but the nurses could promise not to inform the children on their own initiative.” *[26]

The fear of ‘opening up a can of worms’ and being caught between family and personal values led to professionals feeling out of their emotional depth when facing dying parents with young children.

#### Out of their emotional depth

Professionals found it difficult to imagine the child growing up without a parent [20, 30, 31]. The youngest children, in particular, seemed to tug at heartstrings [25]. For those who had prior experience of loss in their own childhoods, close associations with children in their personal lives (e.g. being a parent to a young child) or that related to the situation in other ways, these types of interactions could elicit countertransference, making it harder for them to cope [20–22, 25, 27, 31].

*“The distress was intensified when HP (health professionals) identified themselves with the patients’ situation. HP considered death too sensitive to talk about, because they were confronted with their own mortality, especially when HP were of the same age as the patient…Similarities in age or life situation led the HP to identify themselves with the patients and made them emotionally vulnerable in their encounters. Such identification could show up unexpectedly.” *[31].

Other professionals however leveraged on these experiences to help children by sharing personal stories [20, 21, 23]. While still emotionally difficult for professionals in general, these were instances where their psychological burden was reduced [23]. Professionals had a harder time if they had less experience with children, either professional [19, 24, 27] or personal [27, 31], to draw on.

Nonetheless, all such interactions were described as emotionally draining. Professionals worried about affecting their judgement when making medical decisions [31], especially if they did not have sufficient emotional reserve or were not coping with their own distress [20–22, 31], including concerns over being emotionally attached to the child [24]. Handling emotional issues was left largely to the individual professional, with little or no support provided by either colleagues or the workplace, as part of the underlying work culture [21, 31]. This thread will be expanded later under ‘Potentiating factors within the workplace’. Lastly, sudden changes in the parent’s medical condition that limited the professionals’ time with the child and family added to the quagmire of emotions.

These emotions could result in a sense of helplessness, which led some professionals scrambling to find ways to cope, like excluding children from conversations [30], choosing not to think about them [21] or even sometimes deliberately bringing them up in conversations [23]. These measures prevented professionals from feeling overwhelmed and safeguard medical decision-making [31]. Understandably, greater experience communicating with children reduced distress. Though painful, these encounters in many ways gave meaning to their work. Anecdotally, some were able to address unfinished businesses from their own losses [21].

### Assumptions and actions

The assumption that children were not their responsibility and that it was futile to engage them was pervasive, leading to professionals passing the responsibility onto others.

#### Children are not their responsibility

Professionals felt their responsibility did not include caring for the children [31], partly because they felt they had nothing to offer [24], and also because they felt their role did not include counselling parents regarding their children [21, 22]. Rather, they thought parents were the best people to tell the child what was going on since they knew the child best [23, 32], and that parents *“need to take the main responsibility for the children's health when one of them is in hospital.” *[24].

However, others felt their role was to “empower” parents to have conversations with their children regarding end-of-life issues [23, 32]. They offered suggestions on what to say if the parent asked but left the responsibility of telling the child to the parents [17, 22–24]. They also expected parents to deal with questions the children might have, and to cope with the situation in general [32]. It was deemed appropriate for children to interact with doctors, medical social workers [26] or even mental health specialists only if they had behaviour issues [25]. By delegating this aspect of the child’s care to others, the professional maintained a distance from the children and protected themselves [23]. Apart from assuming that children were not their responsibility, there was also a sense that it was futile to engage them.

#### It is futile to engage them

This belief arose from professionals’ preconceived notions about children and not fully appreciating their own role. There were professionals who thought children would not understand what was going on, and that death was *“something that children could not be prepared for”* [15, 25, 32]. Children’s disruptive behaviour was mostly accepted or quickly dismissed; it was only perceived as pathological if it was deemed not age appropriate. This might have led to missed opportunities for rendering support or legitimised practices that removed ‘disruptive’ children from delicate situations [25]. In addition, as they often took cues from their parents, children were less likely to engage with professionals [18, 19, 25], particularly if parents were reluctant to talk or appeared stoic. All these assumptions, coupled with self-devaluation of the role they played in providing a listening ear and being emotionally present [21], might have led to professionals passing the responsibility of supporting children onto others.

#### Passing the responsibility

Professionals handed over support of these children to other professionals either within their own organisations or outside. They sought specialists deemed more experienced [16] in communicating with children such as social workers [16, 18, 32, 33], chaplains [18, 20], counsellors [18, 24, 25], psychologists [24, 25] or psychiatrists [25, 29, 32]; external community services [22]; or just left the children to their own devices [32]. Other avenues of support included schools [25, 32, 33] and support groups [18, 28]. There were instances where social workers referred children to palliative medicine specialists as these professionals were perceived more capable in dealing with grief matters [16]. One drawback cited about referrals to external counsellors was that service might not be available after hours [20]. Long waiting lists for other services also meant existing professionals had to meet the need themselves [18] in the interim.

Professionals in different settings had differing opinions on who supported these children best. Institution-based professionals thought community services had relatively more opportunities and time [22]. After all, serious or sensitive conversations were assumed easier in a non-clinical setting, where the professional likely knew the family prior to the parent’s terminal diagnosis. Additionally, these community services were perceived to act as conduit between family and hospital for continuity of care, including follow up support after bereavement [15, 27]. Community-based professionals on the other hand believed the acute or institutional setting provided much better support to children [22].

By referring the case on, the professionals remained within their comfort zone [16] as they found supporting children challenging [24]. There is however a caveat: *“It hence represents a risk that no one assumes the duty and responsibility to follow-up on the child”* [25]. Rather than referring cases on, some have suggested that different services collaborate to provide more holistic care [16]. For successful partnerships to happen, potentiating factors within the workplace need consideration.

### Potentiating factors within the workplace

The workplace influenced the overall experience of having serious conversations with children along three factors: work culture and relationships; infrastructure, systems and workflow; and cracks in interagency collaboration.

#### Work culture and relationships

Organisations' protocols and guidelines implying that children were not a priority resulted in professionals feeling alone and unsupported, undervaluing related child-centred work. Professionals mentioned getting a sense (either directly or indirectly) that the adult patient was their focus, and that interacting with their children was not important [16, 19, 21–26, 31, 32].

*“In principle, there should be a patient—that is, a person with a diagnosis—in order to claim professional attention in the medical field … Partners and children do not have a diagnosis related to palliative care and so they do not have the legitimate (medical) right to attention from a doctor or other professionals.” *[32]

Professionals were torn between predefined job scopes, where there was a tacit understanding of whose duty it was to manage the children and competing responsibilities of clinical work, admin duties and research [32]. In some institutions, children were seen as more the responsibility of the nurses, and to a lesser extent, the doctors. The latter were likely to speak to children only if specific issues needed addressing [25].

Confiding in colleagues was one way of coping with intrinsic challenges. However, professionals felt uncomfortable, as they perceived that their workplace did not encourage sharing and lacked an outlet for expressing their struggles [21, 22]. They valued support from colleagues (especially those with more experience) [23], receiving feedback [20, 21, 24] and channels to reflect on interactions [20, 23, 24]. They also expressed desire for role models or mentors for guidance [21, 23], formal clinical supervision [16, 21, 23] and regular facilitated sharing with colleagues in a safe environment [20–23]. This support was viewed positively as it allowed professionals to receive validation for their emotions and seek counsel [20, 23], but oftentimes subjected to limitations imposed by workplace infrastructure, systems and workflow.

#### Infrastructure, systems and workflow

Particular issues raised: training on how to communicate with children, gaps in medical records documentation to transcribe pertinent information about the children, dedicated time to engage them, lack of a child-friendly environment and issues with care continuation (and transfer) post-bereavement.

Professionals considered learning to communicate with children important. Prevalent desire for training on: stages of child neurocognitive development, how children grieve, and how to communicate with them about death and dying in an age-appropriate manner [16, 18–20, 23, 24, 26, 30, 31]. The importance of such training was not always recognised by superiors or the organisation, that either accorded less priority for this skillset in an adult service, or advised onward referrals to better qualified external practitioners [16, 17, 21, 31]. One study participant described what would happen if they asked for relevant training:

*“I need some training on child bereavement issues”, they would say “what for, you are an adult service” … “well you know I’ve got relatives whose kids need help” and they would say “well refer them to social workers, let the teachers know, refer them to the children’s community Macmillan nurse” *[17]

Lack of knowledge made the professionals reluctant to engage with children [16, 17, 24, 31]. Other than training, it was suggested that official guidelines would be helpful [21, 23, 24], as well as education in stress management and self-care [21]. For charitable organisations like hospices, funding constraints have unfortunately impeded training provision to staff [18].

There were instances where medical record systems did not have specific places to document the presence of children or issues relating to them, and so pertinent information got lost in myriad medical data [18, 31]:

*“Interviews with HP (health professionals) showed that they did not register patients’ children systematically with their age, names and needs and that the design of the record system was an obstacle. Thus, HP often lost track of whether the patient had dependent children or not. Nurses had a place in their record called “mental and social” where they sometimes wrote information about children … doctors and nurses registered comments in different systems, so information about children was seldom coordinated or shared and often sank without trace.” *[31]

Routine screening did not include queries about children, and often depended on the professional’s initiative [15, 19, 23]. At times, professionals were not even aware that their patients had young children [19, 20] or the nuances of what was discussed about them previously as details had not been recorded [18]. One way of overcoming this was by sharing at multidisciplinary meetings [18, 20].

Lack of a child-friendly environment in institutional settings as well as visiting hours clashing with school time compounded children’s absence or lack of participation. [19, 21, 24, 26, 27]. Some professionals *“highlighted hospitals as an unsuitable environment for children and talked about the importance of facilitating children’s play so that children could be ‘themselves’ in their own arena – for example, by providing home visits and playrooms in hospitals.” *[25].

Lack of time to interact with children was frequently highlighted. Reasons provided included manpower shortages [21] and work timetable inflexibility when setting time for children [15, 17, 20, 23, 24, 26, 31, 32]. Ideally, support for children should be rendered outside school hours so that they would not have to take time off from school [15, 16, 32], but this would impact staff availability [18]. Overall, having* “more time”* [18, 26] to attend to the children’s needs was deemed important as *“nurses described being overworked and not having the necessary time for the children, making it easy to forget their responsibility for child relatives” *[24].

In instances where social workers were involved prior to bereavement, there were issues regarding continuation and transfer of care. Hanging on to existing clients could compromise capacity to handle new ones, and alternative default providers like General Practitioners might not provide the same level of service [33]. While social workers remained valuable sources of information, their high workload made it difficult to share knowledge across the inter-disciplinary team [33]. Lastly, continuation of care was complicated by observed cracks in interagency collaboration.

#### Cracks in Interagency collaboration

Communication between agencies lapsed when it came to the children, leading to confusion as to who was meant to support the children [19, 20]. The presence of children was either not highlighted [18–20] or key information omitted [29], which resulted in some children not receiving support or lost to follow up [18, 29].

Professionals were unsure what services other agencies provided, and how they might complement their own [21]. Referrals could be dropped [19, 21] if process was unclear or information not signposted [20, 23]. Without follow up, professionals upstream were unaware what happened to the children after bereavement [27, 32], including whether needs were adequately met [22]. Stakeholders advocated for *“flexible services to enhance family commitment to support; expanded types of support before parental death; greater provision for children before and after parental death; greater community- based support; and greater involvement of voluntary support.” *[18].

Not addressing this rendered the children “invisible” [18, 19, 23, 25, 31], resulting in caring professionals overlooking their needs altogether:

*“Children's visibility relies on HPs (health professionals) being aware of the presence of patients’ children and other psychosocial aspects related to this … Of paramount concern to HPs were children who were most likely to go undetected, for reasons such as their parent's limited psychological capacity to advocate for support for their children, or their lack of physical capability to access resources. HPs reported there are some parents with a comorbidity of serious mental and/or physical health problems alongside their cancer diagnosis, and their children are more likely to go undetected.” *[19]

As such, inter-agency collaboration was essential for family-centric care [25]. Ready access to information on available services facilitated such collaboration [22].

## Discussion

In reviewing the existing literature, the extent of discomfort faced by professionals, and its ripple effect on the care of a child with a dying parent was striking. A sense of helplessness stood out, which seemed worse when professionals had less experience or little support from colleagues within an incompatible system and work culture [34]. Personal coping strategies included professional distancing and use of euphemisms. Having professional experience to draw on, workplace support to manage the emotionality [35] and avenues for training as well as self-care helped [36]. Other gaps at agency and organisational levels within the healthcare system were highlighted for their implications on good family-centred care. It appears professionals’ sense of helplessness (and compassionate care) could be addressed through multiple solutions at the workplace.

Professionals tended to acquiesce to a parent when there were competing viewpoints. For example, if a parent withheld information about their illness from their children or excluded them in other ways, the professional complied tacitly. This was prevalent especially where professionals had little or no training. We found disparate opinion on involving the child, ranging from it not being the professional’s responsibility to assertion that it was indeed the child’s right [37]. In some jurisdictions, when a parent has a life-limiting illness, there are in fact laws mandating the rights to information for the minor as next of kin [25, 26, 31, 32]. An apparent lack of awareness among professionals suggests the need for wider educational efforts [25]. A related issue is appropriateness for the professional to expect a parent to take the lead in updating the child. Children like to know about their parent’s condition and think healthcare professionals are a better source of information than their parents [8]. Children are in considerable distress when faced with the imminent death of a parent, and can experience a variety of thoughts, emotions and behavioural issues. Appropriate intervention by a professional, ideally before the parent dies, could help children cope much better [38].

Parents undergo considerable emotional upheaval themselves; the sick parent adjusting to dual roles of being a patient with shortened life expectancy and parent of a young person, while the well parent takes on the role of caregiver as well as expanded responsibilities surrounding the child’s wellbeing [8, 39–44]. Patients with cancer who had young children expected professionals to advise, but found they often had to initiate the discussion [8, 41, 42, 45]. After bereavement, some surviving parents realised they had missed critical information about prognosis, missing the chance for their children to say goodbye or complete important conversations with the deceased. This raised the need for clear and upfront conversations, so families know what to expect, as sudden unanticipated bereavement brings considerable turmoil and disruption to the family [46].

Organisational protocols and staff resources may need to be reviewed to include guidelines and support for professionals as they communicate with young children. In our review, we found that current structures may incentivise professionals to prioritise the patients’ needs, but at the cost of rendering their dependents invisible. To address this, support for these cases should consider the parent and the child as an interdependent dyad, where the impact and well-being of one party directly affects that of the other. An actor-partner interdependence model (APIM) might be useful as the basis for organisations to structure and accommodate for young children [47].

Professionals covered in this review suggested few changes on the individual level, with scarce mention of self-care. There was more robust discussion however, on what might be helpful on an organisation or system-wide scale. Professionals perhaps were uniformly keeping a professional and emotional distance. We found it surprising that provider burnout was not mentioned. The imperfect continuity of care, lost opportunities for feedback and lack of personal closure in bereavement that in our opinion could have coloured overall experience also did not surface. There appears much we still do not know about the experiences of the professional. It is now clear though where and how organisations could support professionals better. Formal processes and structural elements do reflect the workplace’s priorities, like how information is recorded or transcribed and what child-related information is considered important. These components in turn translate to a work culture and ethic that is both receptive and protective to professionals, which ultimately improves support to clients.

Professionals would benefit from learning skills to help them interact confidently with children, as well as having platforms to solicit advice, receive support and express their emotions. Since they spend significant amount of time at work, the workplace would be an ideal location to provide and receive compassionate guidance. Regular meetings between professionals from different institutions who manage such children would help raise awareness on scope of services offered, provide networking opportunities and foster effective collaboration.

Finally, as individual hospices may not always have the capacity to support these children, one solution is to improve the skills of community partners such as schools. Education and training will be needed and hospice workers have been proposed as suitable trainers [48], but this review posits that these same professionals may need to better equip themselves first through relevant experience and training.

Findings potentially inform training that may be supportive to professionals and indicate ways the healthcare system could respond in fostering more effective interactions.

### Limitations

Most articles did not specify the ages of children, but instead used descriptors such as ‘young children’, ‘dependent children’ or ‘minors’. Some articles may thus have been overlooked. Given that children are known to have distinct cognitive-developmental stages, it would be prudent for future empirical studies to refer to children by age or developmental stages (such as Erikson’s Stages of Psychosocial Development [49]) to clarify context.

Another limitation is that the demographic makeup of the participants was more skewed towards nurses. Nurses made up the majority of participants, with doctors, social workers and allied health workers making up smaller numbers. While at the outset we aimed to seek opinions of spiritual care professionals, no relevant studies were found despite the search strategy incorporating this. Moreover, most participants were female. It is thus unclear if findings may be different otherwise. Five studies had not mentioned participants’ designations [15, 16, 18, 28, 33].

The dearth of positive experiences reported by professionals might have been due to the way participants’ opinions were obtained. As most articles reviewed did not include an interview guide, we cannot comment if questions posed had influenced this significantly.

The bulk of the literature came from Western dominant societies, with only one article from Asia (Japan). While we believe cultural context plays a role, this cannot be confirmed based on current literature. We know however that culture influences concepts and understanding around autonomy and disclosure of terminal diagnosis [50].

### Further Research

We identified areas for further research. Firstly, those that revolve around the individual professional. It is still unclear the extent countertransference affects the professional in these situations. Appreciating what more can be done at an individual level would be helpful, as outcomes from systemic changes may take time [51, 52].

Secondly, changes to systems or policies will also benefit from further research. Changes which may be incorporated into routine palliative care workflows include: processes for early identification of patients with young children, allocating more time to attend to such families and providing more supervision and training to staff. As these changes will be facilitated by relevant policy drivers including funding support, they will benefit from further research, with progress tracked over time, and possibly utilising mixed-method study methodologies.

Lastly, longitudinal research on triads of sick parents, their minor children and the professionals throughout the illness journey while under hospice care can reveal how stakeholders interact and make sense of circumstances at different stages. This may reveal novel ways to approach important interactions in question.

## Conclusion

Healthcare, social and spiritual care professionals approach young children of their terminally ill patients with trepidation, discomfort, and feeling out of their depth. They bring personal values, beliefs and perceptions, that together with their own life experiences shape the manner they render support. Lack of experience, training, and support from colleagues and management adds to their discomfort. Ultimately, the professional does not work in silo but alongside others in a helping organisation situated within a larger system. Many dimensional and inter-woven issues raised in this review (depicted in our conceptual model) will benefit from multi-faceted solutions.

## Supplementary Information

**Additional file 1.** 

## Data Availability

The datasets used and/or analysed during the current study are available from the corresponding author on reasonable request.
