# Supplementary material for: Communicating with young children who have a parent dying of a life-limiting illness: a qualitative systematic review of the experiences and impact on healthcare, social and spiritual care professionals
Source: BMC Palliat Care. 2022 Jul 12;21:125. doi: 10.1186/s12904-022-01007-1 (PMC9277932; doi:10.1186/s12904-022-01007-1)
Supplement: Supplementary file 1 — Additional file 1. [file 12904_2022_1007_MOESM1_ESM.pdf]

Table 2. Articles selected for review

AUTHOR; YEAR	COUNTRY; SETTING	PARTICIPANTS	STUDY METHODS	MAJOR FINDINGS
**Alexander, 2020** [19]	Australia; Comprehensive Cancer Centre at a metropolitan tertiary teaching hospital	15 oncology professionals (3 cancer nurse coordinators, 1 nurse practitioners, 3 clinical / oncologist specialists, 2 clinical psychologists/ psychiatrists and 6 psychosocial support workers or other allied health workers)	Semi-structured interviews. Analysis using grounded theory	Poor visibility of children due to factors in clinical environment (e.g., not detected as part of routine screening) and psychosocial factors (e.g. parents shield child from Professionals to protect them, religious / cultural factors, parents' ability to support child, child reluctant to share their thoughts and feelings).
**Arber, 2018** [23]	UK (South of England); 1 acute hospital trust in the acute hospital (inpatient) setting.	13 nurses	Focus groups. Thematic analysis	Emotionally challenging (e.g. identify with parent) so avoid interacting with children; lack of time; child seen as a private domain under purview of parent hence may not want to seem interfering and also expect parent to communicate with child about diagnosis / prognosis; importance of peer support with opportunities to reflect; need for training on communicating with children of different ages.
**Clipsham, 2015** [20]	UK; 1 Hospice inpatient unit	10 hospice nurses	Semi-structured interviews. Thematic analysis.	Children need to be identified at point of referral; multiple barriers to supporting children (lack of time or child-friendly space, parents blocking interaction between child and Professional, uncertainty on which Professional should support child); impact on Professional (communicating with children causes anxiety, reminded of own losses of loved ones, emotional burden, availability of resources and training and need for personal support for Professional).
**Cockle-Hearne, 2020** [18]	UK; 130 Adult hospices	Hospice staff who were deemed appropriate to answer – designations of respondents not mentioned	Web-based, cross-sectional survey, with closed and open-ended questions. Descriptive and non- parametric statistics and framework analysis.	Lack of formal process in documenting presence of children; parents restrict access to children; barriers to access such as distance from service; cultural and spiritual beliefs; difficulty catering to children of different developmental stages simultaneously; lack of funding; emotionally challenging.
**Dencker, 2017** [31]	Denmark; 3 hospital wards	9 doctors and 15 nurses working in haematology, gynaecological cancer, and neuro-intensive care units	Semi-structured interviews and participant observations. Thematic analysis.	Structural barriers (e.g., lack of space in the medical recording system, professional code, time pressure and lack of training); emotional barriers (e.g. the painful nature of the situation and the perceived need of keeping professional distance); the latter tend to grow when the former are not addressed.
**Dunne, 2005** [30]	UK; 2 National Health Services (NHS) Community trusts	25 registered nurses	Unstructured interviews. Collaizzi's seven stages of data analysis.	Feel inadequate and helpless when dealing with children so will exclude children from conversations; emotional involvement

AUTHOR; YEAR	COUNTRY; SETTING	PARTICIPANTS	STUDY METHODS	MAJOR FINDINGS
				difficult to deal with; lack of training / education on interacting with children.
**Fearnley, 2010** [16]	UK; 7 hospices and specialist palliative care centres, 3 projects managed by UK Voluntary Organizations, staff working directly with children in school (number not mentioned)	20 multi-disciplinary professionals (designations not mentioned)	Semi-structured interviews. Thematic analysis.	Professionals avoid children, thereby missing opportunities to support children. Due to several factors: lack of time, fear of opening a can of worms, lack of training, worried of not being able to cope emotionally. Suggests the importance of support, clinical supervision and training.
**Fearnley, 2012** [15]	UK; hospices and specialist palliative care centres (number not mentioned)	16 professionals (designations not mentioned)	Semi-structured interviews. Template analysis.	Importance of age-appropriate communication with children. Professionals reluctant to talk to children: fear of opening a can of worms, thinking children too young to understand, wanting to protect them, not knowing what to say, lack of time. Community-based professional can leverage on their relationship with family prior to diagnosis to support children.
**Fearnley, 2019** [17]	UK; Several settings (hospital, hospice, Independent Organisation, Primary Care Trust, Voluntary Organisation, school) where professionals worked within palliative care or with children and families who had been bereaved	4 nurses, 5 social workers and 7 other support workers	Semi-structured interviews and field notes. Thematic analysis.	Parent is most appropriate adult to speak to child; professional fear; lack of time/training on communicating with children.
**Golsäter, 2016** [24]	Sweden; 1 County hospital	22 nurses	Focus group interviews. Inductive qualitative content analysis.	Variations in nurses’ perceptions: not their responsibility; parents are responsible for child; others can help child (e.g., extended family); not being able to care for child due to lack of time / knowledge or out of fear; being aware of the children's situation and working to support them.
**Hanna, 2020** [22]	UK; 1 healthcare trust	32 professionals in oncology, palliative and community care (3 doctors, 21 nurse/nurse specialists, 2 social workers, 6 allied health professionals	Semi-structured interviews. Thematic analysis.	Passing responsibility onto other professionals; emotionally challenging; managing issues within family (collusion, denial, ongoing and changing needs).
**Hogstad, 2020** [37]	Norway; 2 public hospitals	3 doctors and 8 nurses	Semi-structured interviews. Combined 3 discourse analytical concepts:	Different perceptions of children: they can sense something is wrong; dependent on parents (who are seen as the experts when it comes to the children); seen as disruptive.

AUTHOR; YEAR	COUNTRY; SETTING	PARTICIPANTS	STUDY METHODS	MAJOR FINDINGS
			discourse, interpretive repertoire and positioning.	
**Karidar, 2016** [26]	Sweden; 2 specialised palliative care units	9 nurses in palliative oncology	Semi-structured interviews. Latent content analysis.	Parents are gatekeepers to child's involvement with Professional. The latter are subject to the workplace infrastructure and systems which prioritised other tasks.
**Karidar, 2018** [32]	Sweden; Specialised palliative homecare service	5 doctors and 5 social workers	Semi-structured interviews. Analysed using Bourdieu's theoretical framework.	Limited contact with children (due to workplace culture, systems and infrastructure); parents determined contact between professional and child.
**Kennedy, 2008** [33]	UK; A community palliative care service	6 health professionals (designations not mentioned) and 6 family support workers.	Qualitative pre- and post-intervention evaluation design. Thematic analysis.	Specialised support services for children need to be accessible, able to take on new clients and be sustainable. Work closely with children before, during and after the parent's death. General Practitioners may not be able to offer such services with the same degree of time and expertise.
**Macpherson, 2007** (Parts 1 And 2) [13,14]	UK (Fife, Scotland); General practice	3 General Practitioners.	Semi-structured interviews. Thematic analysis.	Lack of experience and knowledge on children's needs makes it difficult to support them.
**Niemelä, 2010** [29]	Finland; 1 general hospital psychiatry ward	7 mental health professionals (4 psychiatric nurses, 1 social worker, and 2 mental health nurses)	Narrative individual interviews. Narrative analysis.	Need for inter-team collaboration to leverage on expertise; the importance of child-centric work to support children.
**O'Neill, 2020** [28]	Ireland; 2 cancer support centres	5 facilitators who had a professional health and social care background (designations not mentioned)	Interviews. Thematic analysis.	Parents are afraid to talk to their children about cancer, and value professional support. Professionals need to know how to deal with children's needs, which vary due to age and development stage.
**Otani, 2019** [27]	Japan; hospital (number of hospitals not mentioned)	3 doctors and 17 nurses.	Semi-structured, face-to-face interviews. Thematically analysed in the grounded theory tradition.	Lack of knowledge/opportunity/experience with children; impact on own suffering; unsure how much to intervene with family; family shielding the child from the reality of death; estrangement from family once they leave the hospital.
**Turner, 2007** [45]	Australia; 3 large metropolitan cancer services	24 oncology nurses	Focus groups and individual interviews. Thematic analysis.	Emotionally challenging; lack of education, supervision, and support; challenges working with individual families; fear of making things worse; workplace culture, infrastructure and systems not prioritising supporting children.
